# Integrated Utilization of Sewage Sludge and Coal Gangue for Cement Clinker Products: Promoting Tricalcium Silicate Formation and Trace Elements Immobilization

**DOI:** 10.3390/ma9040275

**Published:** 2016-04-07

**Authors:** Zhenzhou Yang, Yingyi Zhang, Lili Liu, Seshadri Seetharaman, Xidong Wang, Zuotai Zhang

**Affiliations:** 1 Beijing Key Laboratory for Solid Waste Utilization and Management and Department of Energy and Resource Engineering, College of Engineering, Peking University, Beijing 100871, China; zgkdyzz@126.com (Z.Y.); zhyingyi2@163.com (Y.Z.); liu-0806@163.com (L.L.); xidong@pku.edu.cn (X.W.); 2 Department of Materials Science Engineering, Royal Institute of Technology, Stockholm SE 100-4, Sweden; raman@kth.se; 3 School of Environmental Science and Engineering, South University of Science and Technology of China, Shenzhen 518055, China

**Keywords:** waste, sewage sludge, coal gangue, eco-cement, clinker, trace element

## Abstract

The present study firstly proposed a method of integrated utilization of sewage sludge (SS) and coal gangue (CG), two waste products, for cement clinker products with the aim of heat recovery and environment protection. The results demonstrated that the incremental amounts of SS and CG addition was favorable for the formation of tricalcium silicate (C_3_S) during the calcinations, but excess amount of SS addition could cause the impediment effect on C_3_S formation. Furthermore, it was also observed that the C_3_S polymorphs showed the transition from rhombohedral to monoclinic structure as SS addition was increased to 15 wt %. During the calcinations, most of trace elements could be immobilized especially Zn and cannot be easily leached out. Given the encouraging results in the present study, the co-process of sewage sludge and coal gangue in the cement kiln can be expected with a higher quality of cement products and minimum pollution to the environment.

## 1. Introduction

With the rapid development of urbanization and industrialization, various solid wastes accumulate year by year, not only occupying land but also causing serious environment problems. Traditional waste disposal methods, such as pile up or landfill, have proven to be of low efficiency, a waste of resources and even environmentally harmful. The increasing shortage of resources and the negative impact on the environment, seen from a global perspective motivated the present work with respect to finding efficient process route toward waste utilization.

Among these solid wastes, sewage sludge (SS) and coal gangue (CG) are two typical common wastes generated. Sewage sludge is a problematic waste generated from wastewater treatment. The production of dried SS in China is currently eight million tons per year and is still increasing at a rate between 8% and 10% annually [1]. Due to the high content of hazardous substances such as organic, non-biodegradable compounds, bacteria and viruses, *etc.*, the improper disposal of SS could pollute the environment severely. The most common treatment methods in China are agricultural utilization, landfilling, and incineration [2,3,4]. However, the reduced availability of land and the presence of pathogens and contaminants such as heavy metals, micro-pollutants, and antimicrobial agents limit applications of these methods. Coal gangue, as one of the common industrial wastes, is one of the main byproduct from coalmining and beneficiation and accounts for about 15–20 wt % of coal production [5,6,7]. CG stockpiles have reached 4.5 billion tons in China and are increasing at the speed of 750 million tons per year. The traditional disposal method of accumulation is also infeasible due to increasingly stringent environmental regulations. Thus, an environment-friendly and economically viable process to jointly dispose of these two wastes is urgently required.

Cement kiln is well known as an ideal device to dispose wastes, which can completely decompose the hazardous substance such as dioxins and furans due to the high temperature and long residual time [8]. Based on the characteristics of SS and CG, these two wastes may be utilized in the cement kiln for clinker production, where the organic matter can serve as an alternative fuel and the residual inorganic ash can be incorporated into clinker as part of raw materials. Thus, the use of SS or CG in cement kiln offers multiple advantages such as reduced use of primary raw materials, lower fuel consumption and significant reduction in CO_2_ emission. Besides, this integrated activity can also produce additional revenue to subsidize government waste disposal.

A number of investigations have been carried out with a view to utilize SS as a replacement for some raw materials used in cement manufacturing [9,10,11,12]. The main focus in the case of most of these studies has been the impact of SS on the properties of cement such as setting time and compressive strength. However, very few investigations have been conducted on how to combine cement plant with CG and SS disposal and their effect on the clinker properties, such as crystalline structure and crystal formation mechanism when they substitute a part of the raw materials for manufacturing of clinkers. Furthermore, due to the increased public concern with environment risk, it is important to evaluate the effect on the environment during the process of utilization of SS and CG, especially the trace element problem. It is well known that the interphase in the clinker has a high capacity to immobilize trace elements due to its specific structure and the silicate phases can also stabilize trace elements through solid solution [13,14]. In addition, the clay minerals in CG can help stabilize trace elements during calcinations. Therefore, the co-process of SS and CG in cement kiln can be expected to be a promising method for environmental protection. To the best of the knowledge of the present authors, the quantitative analysis of trace elements immobilizing into clinkers during the co-process of SS and CG has not been systematically investigated.

In this regard, the aim of these studies is to compare the characteristics of eco-cement clinker (made with SS and CG substitution) with ordinary cement clinkers synthesized in laboratory environment. The formation mechanism, crystalline phase components and structure, and crystalline micrographs were investigated in this study. The distribution behavior of trace elements during eco-clinker production was also studied. Further, the energy and raw materials consumptions were also evaluated. The present studies are expected to provide a method of integrated utilization of SS and CG in cement industry, which can save energy and resources, and improve trace elements immobilization at the same time.

## 2. Experimental Section

### 2.1. Materials Characterization

The dried sewage sludge pellet and coal gangue samples were collected from a municipal wastewater treatment plant located in Beijing, China. Coal gangue was supplied by power generation plant of Pingshuo in Shanxi Province, China. The results of proximate and ultimate analysis of SS and CG are shown in Table 1. As can be seen, SS had lower ash yield and fixed carbon content than that of CG, whereas it had higher volatile matter contents. The contents of sulfur in CG and SS are at low level, which can effectively increase the amount of liquid phase, resulting in decreasing the melting temperature point and its viscosity. The formation of C_3_S might therefore be accelerated. Furthermore, the calorific value of SS was around 9.35 MJ/kg, which was higher than that of CG (4.82 MJ/kg).

The chemical compositions were determined by an X-ray fluorescence spectrometer (XRF, S4-Explorer, Bruker, Karlsruhe, Germany). The XRF analysis of SS and CG are also present in Table 1. It can be seen that the major components of SS and CG are SiO_2_, Al_2_O_3_, Fe_2_O_3_ and CaO, which is the major components to prepare cement clinker. Besides, SS contains high amounts of alkalis and alkaline earth metal elements. It also should be noted that phosphorus levels in SS are much higher than that in CG. The concentrations of trace elements in SS and CG are also shown in Table 1. It can be seen that the trace elements in SS are notably higher than those in CG, especially Zn and Cu.

**Figure 1:**
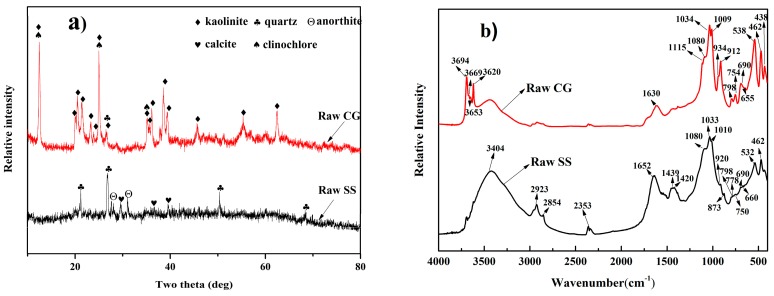
The X-ray diffraction (XRD) patterns and Fourier transformation infrared (FT-IR) spectra of raw coal gangue (CG) and sewage sludge (SS): (**a**) XRD patterns; and (**b**) FTIR spectra.

To understand the structure of solid waste, the SS and CG were analyzed by X-ray diffraction (XRD, D/Max 2500, Rigaku, Tokyo, Japan) and Fourier transformation infrared (FT-IR) and the results are shown in Figure 1. It can be seen from Figure 1a that the major mineral phases in SS are quartz and calcite. In addition, anorthite was also identified in the SS. The XRD patterns of CG show the distinctive peaks belonging to the characteristics of kaolinite. Besides, the mineral phases of clinochlore was also identified in the CG. The FT-IR spectra of SS and CG are presented in Figure 1b. As for SS, the peaks at 3404, 2923, 2854, 1439, 2353, and 1652 cm^−1^ are related to the organic function groups. This indicated the presence of high content of organic material such as carboxylic, alcoholic and amide compounds [15]. The high content of organic material likely lead to a high calorific value of SS compared to that of CG. Besides, the peaks at 1420 and 873 cm^−1^ indicate the existence calcite, while the peaks at 1010, 920, 750, 660, 530 and 462 cm^−1^ are the characteristic bands of anorthite [16]. As for CG, the peaks at 3694, 3669, 3653, 3620, 1115, 1100, 1034, 1009, 934, 912, 792, 754, 540 and 431 cm^−1^ are the characteristic bands of kaolinite while the peak at 3620, 1085, 1630, 1009, 655 and 460 cm^−1^ belong to the characteristics of clinochlore [16,17]. Furthremore, the peaks at 1080, 798, 690 and 462 cm^−1^ indicate the existence of quartz in CG and SS [16]. It can be seen that a strong band at 1033 cm^−1^ can be found in the spectra of CG and SS. For CG, this band belong to the Si–O stretching of Si–O–Si while for SS, it might be attributed to the O–H in mineral components or the existence of C–O function group [18,19].

**Figure 2:**
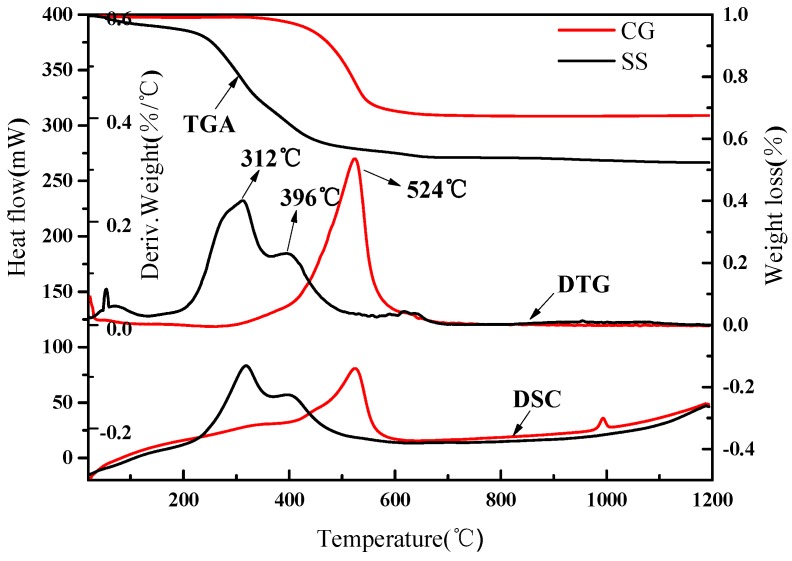
The thermogravimetric (TG), derivative thermogravimetric (DTG) and differential scanning calorimetric (DSC) curves of raw materials.

The thermal analyzer (Q600SDT, TA Instuments, New Castle, PA, USA) was used to study the thermal behavior of SS and CG. The measurements were conducted from ambient temperature to 1200 °C at a linear heating rate of 10 °C/min under air as atmosphere and the results are shown in Figure 2. It could be seen that the most rapid mass loss occurred at around 200 °C and two distinct peaks appeared in the derivative thermogravimetric (DTG) and differential scanning calorimetric (DSC) curves of SS. The former peak (200 to 370 °C) was attributed to the combustion of volatiles and the second peak (370 to 500 °C) corresponds to the char oxidation. On the contrary, the CG showed a different thermal behavior: rapid mass loss occurred in the temperature range between 400 and 600 °C. The DTG and DSC curves showed only one distinct peak. It could also be noted that the spontaneous combustion did not occur until the temperature exceeded 200 °C. This suggested that the SS and CG can easily be stored and handled, without any risk of potential explosion from spontaneous combustion during the manufacturing process when using SS and CG as raw materials [20].

### 2.2. Eco-Cement Clinkers Preparation

The raw meals (raw materials to prepare for clinker) were prepared by mixing CaCO_3_, SiO_2_, Al_2_O_3_, Fe_2_O_3_ and various amounts of SS or CG. All the reagents used here were of analytical reagent grade. The compositional parameters in cement chemistry are listed as Equations (S1)–(S4).

The raw meals were dried in air at 105 °C for 24 h and ground in a ball mill until the particles can pass through #200 mesh metallic sieve. Then, they were prepared under different blending ratios with SS additions of 5, 10, 15 and 30 wt %, respectively. In consideration of cement clinker parameters, the mixing ratio of CG was chosen as 5 and 10 wt %, as shown in Table 2. All the mixture were pressed to φ20 mm × 5 mm slices by applying a pressure of 10 Mpa and calcined in the programmable electrically heated tube furnace. The furnace temperature was raised at the rate of 10 °C/min from room temperature to 1450 °C. The temperature was maintained at 950 °C for 30 min to ensure the complete decomposition of CaCO_3_ and held at 1450 °C for 2 h. After calcination, the eco-cement clinkers were cooled rapidly in air and pulverized to pass through #200 mesh metallic sieve. The samples were collected for XRD, FTIR, and scanning electron microscope (SEM, S4800, Hitachi Ltd., Tokyo, Japan) analyses and the particle size distribution of raw meals are shown in Figure S1.

### 2.3 Trace Element Immobilized Characterization

In order to investigate the transformation behavior of trace elements during calcinations, the samples were digested in microwave assisted digestion system (EXCEL, TOPEX, PreeKem, Shanghai, China) in triple acid (HNO_3_:HF:H_3_PO_4_ = 4:2:1). The concentrations of selected trace elements As, Cd, Co, Cr, Cu, Ni, Pb and Zn were measured by inductively coupled plasma-atomic emission spectroscopy (ICP-AES, Prodigy XP, Leeman, Hudson, NY, USA). Standard reference materials GBW08401 (coal fly ash) [17] and GBW07406 (GSS-6, soil) [21] were used for calibration. The accuracy for the trace elements was within ±10%.

In order to determine the potential leachability of trace elements in clinkers, Toxicity Characteristic Leaching Procedure (TCLP) method of the US Environmental Protection Agency (US-EPA) was applied to the clinker. The extracting solution used was 0.1 M acetic acid at pH 4.93 and the trace elements in leachate were determined by ICP-AES.

## 3. Results and Discussion

### 3.1. Mineralogical Characterization of the Eco-Cement Clinker

**Figure 3:**
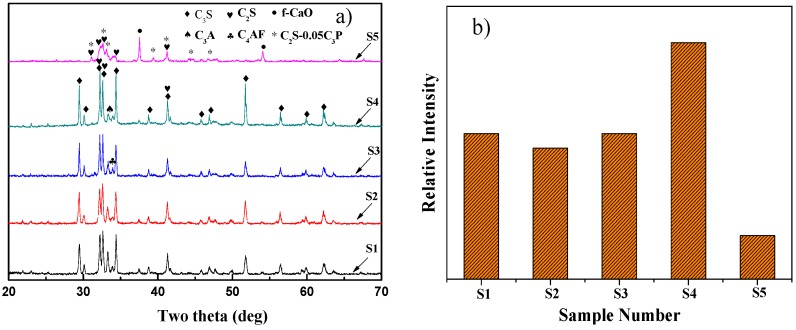
The XRD patterns of the clinker adding with different amount of SS and the peak intensity of C_3_S: (**a**) XRD patterns; and (**b**) comparison of XRD peak intensity at 2θ range from 31.8° to 32.4°.

The XRD patterns of eco-cement clinker with different amounts of SS addition are shown in Figure 3a. It can be seen that the major components of eco-cement clinkers are C_3_S (Ca_3_SiO_5_), C_2_S (Ca_2_SiO_4_), C_3_A (Ca_3_Al_2_O_6_ and Ca_3_(Al,Fe)_2_O_6_) and C_4_AF (Ca_4_Fe_2_Al_2_O_10_). Thus, the synthesized eco-cement clinkers exhibit the characteristics similar to those of ordinary Portland cement. Amongst these components, C_3_S is the major component of Portland cement clinker that determines its quality. The most important characteristic peaks of C_3_S appeared at 2θ of about 32° and the intensity of peak that is indicative of the relative contents of C_3_S in the clinker [22,23] (see Figure 3b). It should be noted that the characteristic peak of C_3_S shows no significant changes with SS addition up to 10 wt %. With further increasing SS addition, the intensity of diffraction peak of C_3_S is found to increase significantly, indicating the positive effect on the formation of C_3_S. However, the C_3_S diffraction peak dropped sharply with higher SS additions of up to 30 wt % and the phases of f-CaO and C_2_S-0.05C_3_P_2_(Ca_3_(PO_4_)_2_) are formed. These results indicate that appropriate amount of SS addition is favorable for the formation of C_3_S, while excessive SS addition shows the opposite effect on the formation of C_3_S.

It is also noted that SS addition plays a significant impact on the C_3_S crystal structure, which is illustrated in Figure S2. For the blank sample S1 without SS addition, the only diffraction peak observed with regard to the crystal structure of C_3_S is between 51.2° and 52.2°, indicating a rhombohedral (R) structure [24,25]. The split blip between 31.5° and 33° indicates the existence of triclinic or monoclinic crystal structure of C_3_S in blank sample. With the amount of SS additions increasing, the split blip became insignificant and the C_3_S showed the total rhombohedral structure. With the SS additions up to 15 wt %, the shoulder peak appeared between 31.5°~33° and 51°~52.5°, which is characteristic of monoclinic (M) structure. Thus, the C_3_S polymorphs show transition from rhombohedral to monoclinic structure with increasing amount of SS addition.

**Figure 4:**
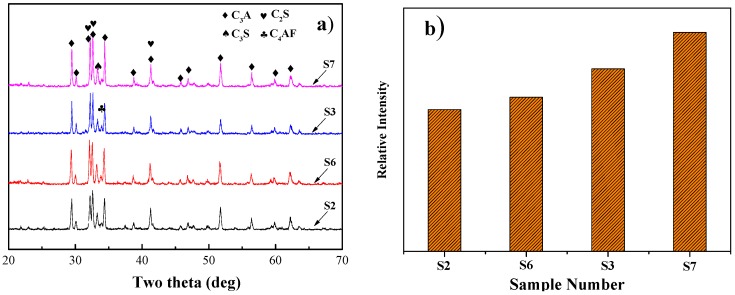
The comparison XRD patterns of the clinker adding with different amount of SS, CG and the peak intensity of C_3_S: (**a**) XRD patterns; and (**b**) comparison of XRD peak intensity at 2θ from 31.8° to 32.4°.

To study the differences of crystal characteristics between clinkers with various amounts of SS and CG additions, a comparison of the crystalline phases formed in the clinkers with various additions of SS and CG is presented in Figure 4. In view of the high content of alumina in CG (28.1%), the maximum amount of CG to be added cannot exceed 10 wt %. It can be seen from Figure 4a that the major crystalline phases of the clinkers produced by the addition of CG are very similar to that of commercially available Portland cement. With a higher amount CG addition, a more intense peak of C_3_S was observed, as shown in Figure 4b, indicating that addition of CG favor C_3_S phase formation. Moreover, it is also noteworthy that the intensity of C_3_S characteristic peak with CG addition was higher than that with SS addition. The results might indicate that addition of CG is more favorable for the formation of C_3_S in the clinkers compared with SS addition. In addition, as shown in Figure S3, the characteristic diffraction peak between 31.5°~33° and 51°~52.5° show clearly that the structure of C_3_S was rhombohedral (R), which indicates that addition of CG has very little impact on the C_3_S crystal structure.

Compared to blank sample S1, the positive effect on the C_3_S formation with CG addition may be attributed to the special structures of CG. The mineral phase of CG consists mainly of kaolinite, which is weakly bonded with hydroxy group. During the process of calcinations, CG is likely to generate a large amount of active silica and alumina, which can then react with calcium oxide to promote the formation of C_3_S. Meanwhile, the hydroxyl group is also broken, and the released oxygen may attribute to depolymerizing Si–O within the raw meals [26,27].

### 3.2. Structure Analysis of the Eco-Cement Clinker

**Figure 5:**
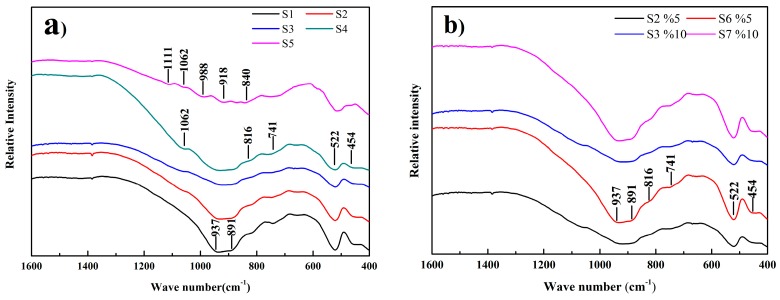
FT-IR spectra for the eco-cement clinkers: (**a**) clinkers with different amounts of SS additions; and (**b**) clinkers with different amounts of SS and CG additions.

In order to further identify the mechanism of phase generation and the structure transformation, clinkers with different amounts of SS and CG additions were analyzed by FT-IR spectra. The results obtained are shown in Figure 5 and the detail peak assignments are presented in Supplemental Table S1.

The most intense peaks at 937 and 891 cm^−1^ and the shoulder at 816 cm^−1^ could be attributed to the characteristic of C_3_S [28]. Meanwhile, the peak at 741 cm^−1^ can be attributed to the Al–O vibrations in C_4_AF while the peak at 454 cm^−1^ correspond to the Al–O vibration at C_3_A [29] is indicative of the existence of C_3_A and C_4_AF in the clinker. For the sample S1 without SS addition, the peaks at 937 and 891 cm^−1^ are separated. With SS addition up to 10 wt %, the two break peaks at 937 and 891 cm^−1^ merged into a single peak, and the peak at 816 cm^−1^ became less profound. This indicates that the crystal structure of C_3_S transformed from a relatively poor symmetry structure to a more symmetrical total rhombohedral structure. The peaks at 937 and 891 cm^−1^ became more distinct again and the peak at 816 cm^−1^ reappeared as the SS addition was increased to 15 wt %, indicating the appearance of monoclinic structure in C_3_S. Furthermore, the peaks of 937 and 891 cm^−1^ became more profound, indicating the higher contend of C_3_S to some extent. With the SS addition at 30 wt %, the characteristic peaks of the C_3_S became less intense and the peaks at 988, 918 and 840 cm^−1^ appeared. The three peaks correspond to the Si–O vibration of C_2_S [30]. Besides, the peaks at 1062 cm^−1^ indicated the existence of (PO_4_) group. The results demonstrate that the decomposition of C_3_S and the formation of C_2_S solid solution, which conform to the XRD results (Figure 3a).

Figure 5b shows the differences between the eco-cement clinkers with simultaneous addition of SS and CG. It could be seen that the clinkers kept the C_3_S structure with CG addition but there were some differences. The intensity of C_3_S characteristic peaks with CG addition is more profound than that with SS addition, indicating the formation of C_3_S with CG addition. Moreover, the results also show that there has no obvious modification effect on the C_3_S polymorphs with different amounts of CG addition and its crystal structure is more asymmetric compared to the one with SS addition.

### 3.3. Liquid Phase Formation during Clinker Calcination Process

**Figure 6:**
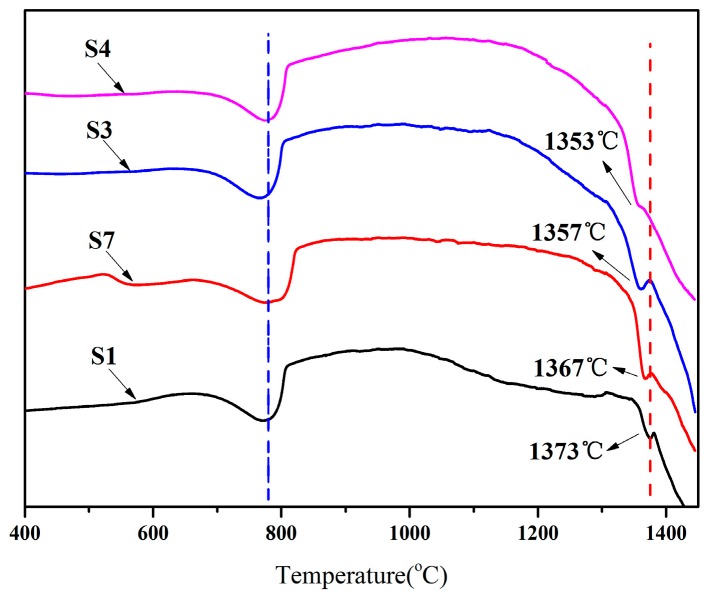
DSC curves for raw meals during heating process.

DSC experiments were conducted to examine the influence of SS and CG additions on the thermal behavior during the clinker formation and the results are shown in Figure 6. It can be seen that the first peak occurred at approximately 785 °C corresponding to the decomposition of CaCO_3_ and the second peak appeared at temperature between 1350 and 1400 °C indicating the formation of liquid phase. The results reveal the fact that addition of SS and CG has no significant effect on the decomposition of CaCO_3_ but can lower the temperature of liquid phase formation, which can be attributed to the existence of trace elements, such as Cu and Zn in the SS and CG (Table 1). The impurities would reduce the melting point of local area melt and favor for the formation of liquid phase during the process of calcinations [31,32]. The chemical reaction between C_2_S and CaO could be accelerated with increasing liquid phase. Besides, the elements of magnesium and sulfur introduced by SS or CG can also lower the viscosity of liquid [33]. Therefore, the additions of SS and CG are likely to boost C_3_S formation and save energy consumption to some extent.

### 3.4. The Immobilization Behavior of Traces Elements

**Figure 7:**
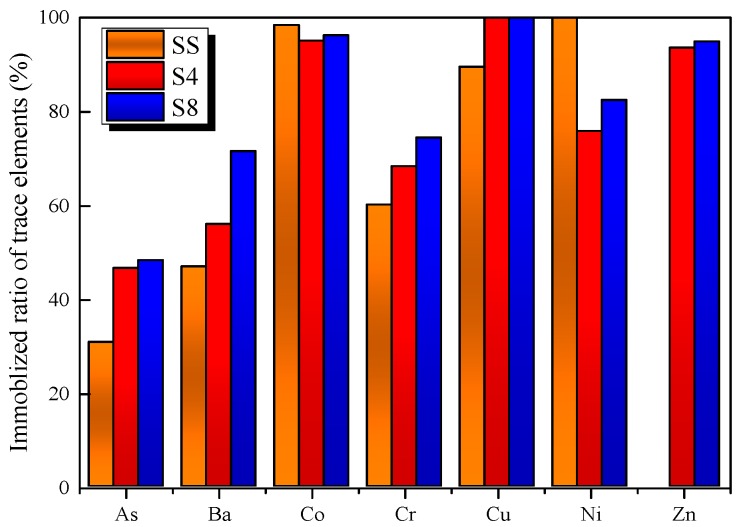
The immobilized ratio of trace elements in ash/clinker during the calcinations process of raw SS, sample S4 and sample S8.

Trace elements have received more and more attention due to their hazardous effect on human health and ecosystem [34,35]. The concentration of selected trace elements in the ash of SS and the eco-cement clinkers were analyzed and the following equation can be used to describe the immobilized effect of the trace elements [36].

$$G=\frac{K}{\frac{S}{1-L}}$$

where *G* is the immobilization ratio of trace elements; *K* represents the trace elements concentration in clinker (μg/g); *S* stands for the trace elements concentration (μg/g) in raw meals; and *L* is the ignition loss (%) after burning the raw meals at 1450 °C. Figure 7 illustrates the trace elements immobilization ratio in ash/clinker during the calcinations process of raw SS (samples S4 and S8). Due to the low concentration of Cd in SS and CG, this element could barely be detected in both raw meals as well as clinker. On the other hand, Pb showed high concentration in raw meals. However, the concentration of lead was found to be below the detection limits in the ash and the clinker, which could be due to the high volatility. Hence, only distribution of As, Ba, Co, Cr, Cu, Ni and Zn are discussed in this part.

For raw SS, it can be seen that Co, Ni and Cu are non-volatile and are mainly enriched in the ash, while As, Ba and Cr are enriched in the ash to different degrees and are classified as semi-volatile elements. Furthermore, it should be noted that Zn is recognized as a highly volatile element in SS and ended up in the gas phase during the calcination process at 1450 °C. This is consistent with previous study showing that Zn showed high volatility due to the high content of chlorine in the SS [37]. For sample S4 with 15 wt % SS addition, it can be seen that the raw meals do have the immobilization effect with respect to most of the trace elements in SS during the burning process, especially Zn and over 90% of Zn can be trapped in the clinker. It is known that the mineral phases in the clinker has a high capacity to immobilize trace elements. Zn, As, Co, Cu and Ni can exist in both of interphase and silicate phase while Cr is preferentially found in the silicate phase [14,38]. Thus, the high retention of Zn can be ascribed to the formation of oxidic solid solution in the mineral phase of clinker during calcination. This phenomenon was also observed by Barros *et al.* [39].

In order to immobilize trace elements of the SS in the raw meals to a greater extend and avoid vaporization of volatile elements, SS should be blended with CG as additive into raw meal. The maximum amount of CG addition was recommended as 5 wt % in consideration of cement clinker modulus, as shown in sample S8. It could be seen that, with the CG addition, immobilization ratio of almost all the trace elements increased as compared with sample S4. The elevated retention effect of trace elements can be attributed to the specific structure of kaolinite. During the calcinations, the crystalline structure of kaolinite is transformed to semi-crystalline metakaolinite and further transformed to mullite. During the reaction series, the decomposition and transition of the crystal structure results in a charge imbalance and the elements may be chemically bonded to the aluminosilicate structure [40]. Therefore, the addition of CG can facilitate the prevention of trace elements emission.

The trace elements incorporated in the clinkers might be leached out under acidic conditions and cause damage to the environment. To investigate this possibility, the toxicity characteristic leaching procedure (TLCP) was conducted to identify the toxicity of the clinker. The regulatory threshold values in china are in accordance with GB 5085.3-2007 (identification standards for hazardous wastes-identification for extraction toxicity of China) [9]. Table 3 shows the leaching concentration of toxic elements and the limit of the extraction procedure toxicity standards in china. For the cement clinker with SS addition (sample S4), it can be seen that only Ba and Zn are present in the leachate and none of them exceeded the regulatory thresholds. This indicates the immense immobilization effect of clinker mineral for trace elements. In addition, no big differences on the leaching ability of trace elements could be noticed with the addition of CG into the clinker (sample S8) except for Zn and Ba. The addition of CG caused a little higher concentration of Ba but lower concentration of Zn in the leachate. The leaching experiment results show that the eco-cement clinkers are safe to the environment.

### 3.5. Energy and Material Balances with SS and CG Addition

The previous study indicated that one tonne of Portland cement clinker production need 3–4 GJ primary energy consumptions and 1.6 tonne of raw meals supplement, respectively [41,42]. It could be seen from Table 1 that the calorific value of SS is equivalent to 9.35 GJ/t. According to the present results, 15 wt % the raw meals could be replaced by SS (0.24 t), *i.e.*, SS can supply about 2.25 Gt energy during the process of calcinations. Under this condition, the fuel consumption in the manufacture of cement could be reduced by more than 50% with 15 wt % SS addition. Further, for the purpose of immobilizing more trace elements, the blend of SS and CG could be added into raw meals as mentioned above (sample S8). According to the present calculation, about 2.5 Gt energy can be saved, which could reduce the consumption of energy above 60%.

**Figure 8:**
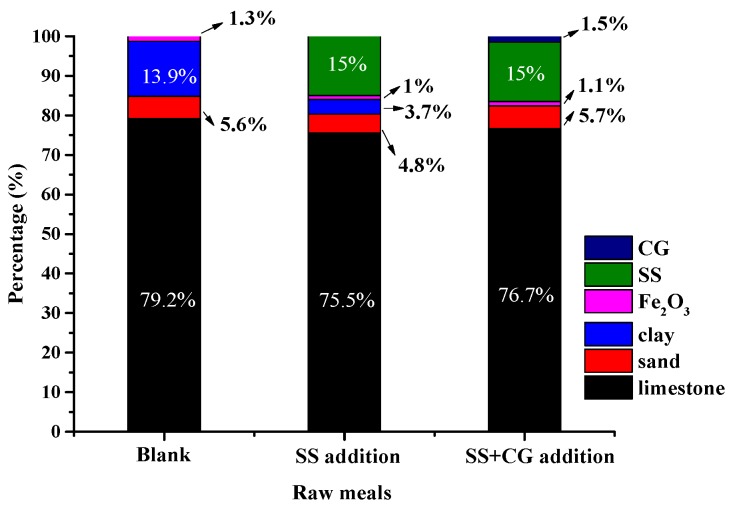
The impact on raw meals compositions with SS or the blend of SS and CG addition.

Furthermore, it could also be calculated that incorporation of SS at 15 wt % in raw meal would result in reducing raw materials requirement of CaO by approximately 8.1 kg/t raw meals, Al_2_O_3_ by 1.1 kg/t raw meals, SiO_2_ by 1.1 kg/t raw meals and Fe_2_O_3_ by 0.8 kg/t raw meals. The raw meals are traditionally extracted from natural materials including clay, limestone, sand and iron powder. Assuming that using the same nature materials in reference to produce clinker [21], the chemical analyses of the raw materials are shown in the Table S2. Figure 8 illustrates the change of compositions of raw meals made by the nature materials by SS and CG additions. For the raw meals with only SS addition, it can be seen that there will be a significant decrease in the raw materials requirement with 15 wt % SS addition, especially for clay. Due to the incorporation of SS, the raw material requirement of limestone would be reduced by 59.2 kg/t clinker, clay by 163.2 kg/t clinker, sand by 12.8 kg/t clinker and Fe_2_O_3(ref)_ by 4.8 kg/t clinker, *i.e.*, the addition of SS can replace approximately 4.7 wt % of limestone, 73.4 wt % of clay, 14.3 wt % of sand and 23.1 wt % of Fe_2_O_3(ref)_. For the raw meals with the blend of SS and CG addition, due to the limit by the modulus of raw meals, the maximum amount of CG addition could only be up to 1.5 wt % when mixed with 15 wt % SS. It can be seen that, with the CG addition, there is no significant decrease in the raw materials requirements except the clay, which can be totally replaced. It is admitted that this would lead to a minor increase in the demand for limestone and sand. Therefore, it is important to take into account the costs of raw materials in the cement manufacturing process and determine the most cost-effective raw material proportion.

## 4. Conclusions

The present results clearly demonstrate that sewage sludge and coal gangue can be used successfully as alternative materials and fuel in clinker manufacturing at the laboratory scale. The main conclusions of the work are summarized below:

1. Appropriate SS and CG addition could be beneficial to C_3_S formation while excess SS addition had the negative effect. The decomposition of C_3_S into C_2_S and f-CaO was found to occur as the SS addition was increased to 30 wt % and the amount of SS blending in the raw meals should be strictly controlled. The positive effect of SS and CG additions for C_3_S formation can be attributed to different mechanisms. The former could be described by introducing a mass of impurities with low melting point, which could lead to decreasing the temperature of liquid phase formation while the special structure of CG could make it easier for the reaction between f-CaO and C_2_S. In addition, the raw meals with CG addition could boost more C_3_S appearance compared with SS.
2. SS could lead to the modification of C_3_S polymorphism from the relatively asymmetric structure to more symmetrical structure (R) with increasing SS addition. As SS addition is increased to 15 wt %, the crystal structure of C_3_S becomes monoclinic (M).
3. It was found that clinkers had a good effect on immobilizing most of the trace elements in SS, especially Zn. CG can also help stabilize trace elements during the calcinations process. The TCLP results show that the eco-cement clinkers met the standards of the current Chinese regulatory thresholds.
4. Integrated utilization of SS and CG as alternative raw materials and fuel in cement manufacture could effectively reduce raw material and energy consumption. They are expected to reduce energy consumption by more than 60% as well as reduce a fair amount of the natural raw material consumption according to the present estimation.

## Figures and Tables

**Table 1:** Chemical compositions of coal gangue and sewage sludge.

Proximate Analysis (wt %)						Ultimate Analysis (wt %)				Calorific Value (MJ/kg)	
-	moisture, ad ^a^	ash, ad ^a^	volatile matter, ad ^a^	fixed carbon, ad ^a^	Total	C, ad	H, ad	N, ad	S, ad	-	
CG	0.8	67.9	15	16.3	100	17.5	12.6	0.6	1.2	4.82	
SS	2	53.3	35.8	8.9	100	23.2	4.0	3.0	1.3	9.35	
Chemical Composition (wt %)											
-	SiO_2_	Al_2_O_3_	Fe_2_O_3_	CaO	MgO	K_2_O	Na_2_O	P_2_O_5_	Others	LOI	Total
CG	32.8	28.8	1.2	0.1	<0.1	0.1	<0.1	0.1	2.5	34.4	100
SS	25.2	5.6	4.2	6.4	1.5	1.2	0.6	4.9	1.4	48.6	100
Trace Elements Contents (mg/kg)																				
-	As	Ba	Cd	Co	Cr	Cu	Mn	Ni	Pb	Zn	
CG	19	ND	ND	8	25	15	6	4	9	14	
SS	50	256	2	11	124	358	543	41	48	1084	

^a^ ad, air dried; LOI, loss on ignition at 1000 °C; Others, other elements; ND, not detected.

**Table 2:** The addition amount of sewage sludge and coal gangue in raw meal.

Sample	Addition Amount (%)	
	SS	CG
S1(Blank sample)	0	0
S2	5	0
S3	10	0
S4	15	0
S5	30	0
S6	0	5
S7	0	10
S8	15	5

**Table 3:** Toxicity characteristic leaching procedure (TCLP) leaching concentrations (mg/L) of eco-cement clinkers.

Sample	Elements (mg/L)								
	As	Ba	Cd	Co	Cr	Cu	Ni	Pb	Zn
S4	ND	1	ND	ND	ND	ND	ND	ND	0.06
S8	ND	2.13	ND	ND	ND	ND	ND	ND	ND
GB 5085.3-2007	5	100	1	~	15	100	5	5	100

ND: not detected, ~: not mentioned in the regulation.

## Supplementary Materials

The following are available online at www.mdpi.com/1996-1944/9/4/275/s1.
