# Supplementary material for: Integrated Utilization of Sewage Sludge and Coal Gangue for Cement Clinker Products: Promoting Tricalcium Silicate Formation and Trace Elements Immobilization
Source: Materials (Basel). 2016 Apr 7;9(4):275. doi: 10.3390/ma9040275 (PMC5502968; doi:10.3390/ma9040275)
Supplement: Supplementary file 1 [file materials-09-00275-s001.pdf]

Supplementary Materials: Integrated Utilization of Sewage Sludge and Coal Gangue for Cement Clinker Products: Promoting Tricalcium Silicate Formation and Trace Elements Immobilization

Zhenzhou Yang, Yingyi Zhang, Lili Liu, Seshadri Seetharaman, Xidong Wang and Zuotai Zhang

1. The Equations of Compositional Parameters in Cement Chemistry:

Lime saturation coefficient:

$$KH = (CaO - 1.65\,Al_2O_3 - 0.34\,Fe_2O_3) / 2.8\,SiO_2 \quad (S1)$$

0.902 < KH < 0.915.

Silica Modulus:

$$SM = SiO_2 / (Al_2O_3 + Fe_2O_3) \quad (S2)$$

2.25 < SM < 2.35.

Iron modulus:

$$IM = Al_2O_3 / Fe_2O_3 \quad (S3)$$

1.3 < IM < 1.4.

Lime saturation:

$$L.S.F = CaO / (2.8\,SiO_2 + 1.18\,Al_2O_3 + 0.65\,Fe_2O_3) \quad (S4)$$

0.94 < L.S.F < 0.96.

2. Prepared Method

All mixtures were pressed to ϕ20 mm × 5 mm slices by applying a pressure of 10 Mpa and calcined in the programmable electrically heated tube furnace. The furnace temperature was raised at the rate of 10 °C/min from room temperature to 1450 °C. The temperature was maintained at 950 °C for 30 min to ensure the complete decomposition of $CaCO_3$ and held at 1450 °C for 2 h.

Table S1. Characteristic bands of FTIR spectra.

Band Center (cm^{-1})	Spectral Feature	Probable Assignment
3694, 3669, 3653	Band	Outer OH stretching
3404	Band	O–H stretching
2923, 2854	Shoulder	Symmetric C–H stretching
2353	Band	O–C=O stretching
1652	Band	C=O stretching
1439	Peak	C=O symmetrical stretching
1165	Band	Trace of quartz
1115, 1110	Shoulder	Apical Si–O stretching
1062	Shoulder	(PO_4) groups
1034, 1009	Band	Si–O stretching of Si–O–Si and Si–O–Al
988	Shoulder	$\nu_3(SiO_4)$ in C_2S
937	Band	$\nu_3(SiO_4)$ in C_3S
934	Shoulder	Al-OH libration (inner, outer)
918	Shoulder	$\nu_3(SiO_4)$ in C_2S
891	Band	$\nu_3(SiO_4)$ in C_3S
840	Band	$\nu_1(SiO_4)$ in C_2S
816	Shoulder	$\nu_1(SiO_4)$ in C_3S
797, 780	Shoulder	Trace of quartz
792, 754, 695	Shoulder	OH translation
741	Shoulder	Al-O vibrations in ferrite and tricalcium aluminate
522	Band	SiO_4 tetrahedra out-of-plane bending vibrations
538	Band	Si–O–Al^{VI} deformation
470, 431	Band	Si–O bending
454	Band	Al–O vibration in C_3A

Table S2. The chemical analysis of the raw materials in the reference.

Raw Meals	SiO_2	Al_2O_3	Fe_2O_3	CaO	LOI
Limestore	5	2.4	0.74	50	40.7
Sand	88.6	2.9	2.67	2.2.	2.61
Clay	37.1	10.6	4.23	18.7	20.6
Fe_2O_3	0	0	96	0	0.5

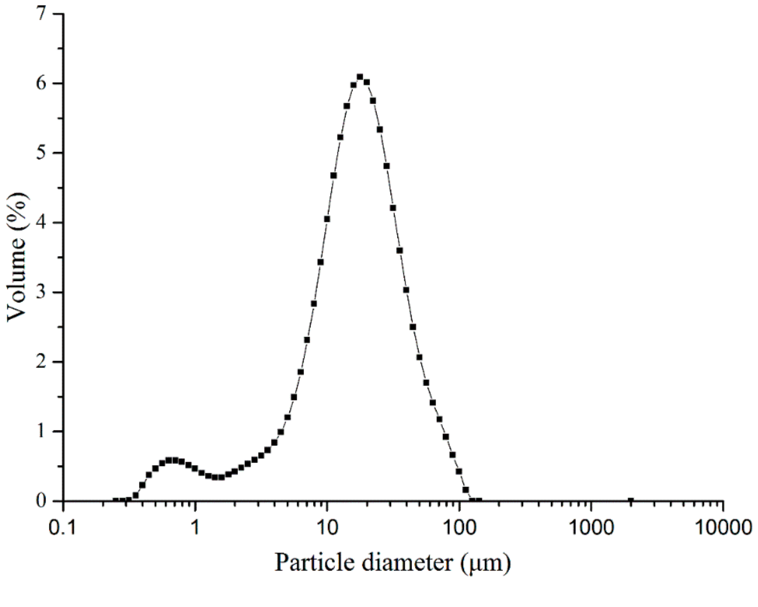

Figure S1. Particle size distribution of raw meals.

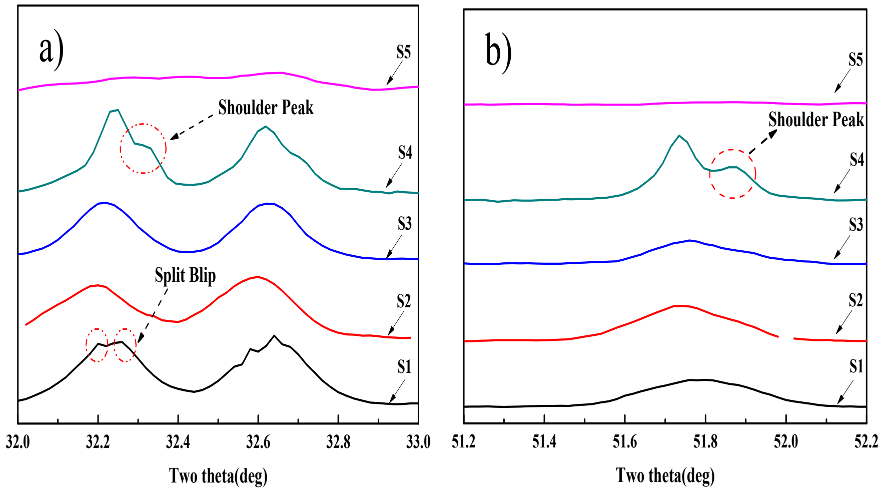

Figure S2. Detail of XRD peaks appearing between 32° and 33°, and between 51° and 52° with different amount of SS addition: (**a**) XRD peak between 32° and 33°; and (**b**) XRD peak between 51° and 52°.

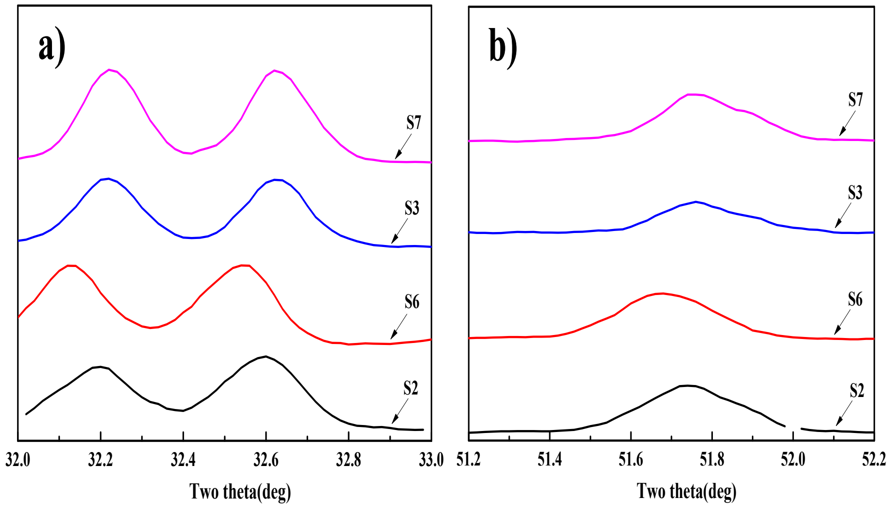

Figure S3. The comparison detail of XRD peaks appearing between 32° and 33°, and between 51° and 52° with different amount of SS and CG addition: (**a**) XRD peak between 32° and 33°; and (**b**) XRD peak between 51° and 52°.
